# Radiographic measurement of the quadriceps angle in dogs

**DOI:** 10.1371/journal.pone.0185833

**Published:** 2017-10-12

**Authors:** Stefania Pinna, Noemi Romagnoli

**Affiliations:** Department of Veterinary Medical Sciences, School of Agriculture and Veterinary Medicine, Alma Mater Studiorum, University of Bologna, Ozzano Emilia, Bologna, Italy; University of Pisa, ITALY

## Abstract

The direction of the force of the quadriceps muscle group is expressed by the value of the quadriceps angle, between the long axis of the muscle rectus femoris and the patellar ligament. This value is often modified in dogs with patellar luxation, a common orthopaedic disease of the stifle joint in both small and large dogs. The aims of the present study were to give a reference value for the quadriceps angle in healthy small and medium-large breed dogs. The medical records of healthy dogs and their pelvic limb radiographs (2014–2016) were reviewed. The radiographs were then divided into two groups: Group A (small dogs <15 kg body weight) and Group B (medium-large dogs >15 kg). The quadriceps angle of each pelvic limb was assessed using a digital software program in order to compare values between the two groups. The radiographs of 160 dogs, 80 per group were studied along with the assessment of the 320 pelvic limbs. The median quadriceps angle values obtained were in contrast with the literature: in Group A, it was 18.3° and, in Group B, it was 8.7°; moreover, the quadriceps angle value for Group A was statistically higher than for Group B (p<0.0001). No significant difference was present between males and females, or between right and left hindlimbs. In Group B, the Labrador Retriever breed influenced the quadriceps angle (p = 0.0005). The outcome of this survey provides an objective parameter, or additional information, to explain the different quadriceps mechanisms of small and medium-large dogs. In future studies the QA range values assessed in the two size groups may be useful in defining the magnitude of the malalignment of the extensor mechanism in both healthy dogs and ones with patellar luxation.

## Introduction

Patellar luxation (PL) is a common orthopaedic disease of the stifle joint in both small and large dogs. Small breed dogs are 12 times more predisposed than large breed dogs to develop medial patellar luxation (MPL) [1]. This statement has been reported in several papers at different times over the last 40 years, i.e. from Trotter [2] and Hayes et al. [3] to Hayashi et al. [4] and Olimpo et al. [5]. The high frequency of MPL in small dogs suggests a hereditary basis and a genetic predisposition [3,6]. The pathogenesis is complex; the patella is part of the extensor mechanism, composed of the quadriceps muscle group, patella, trochlear groove, patellar ligament and tibial tuberosity [4]. Malalignment of one or more of these components creates an imbalance of the quadriceps mechanism forces, which leads to patellar luxation [7].

As described by Kaiser et al., the malalignment in dogs was quantified measuring the quadriceps angle (QA), using radiography and magnetic resonance tomography, and was then related to normal stifle and clinical grades of luxation [8]. The force generated by the quadriceps muscle has an axial deviation of 10° medially; therefore, this angle is referred to as a physiological angle of 10°.

Decreased anteversion, coxa vara, medial displacement of the quadriceps group, external rotation and/or torsion of the distal femur, lateral displacement of the femoral trochlea, internal rotation and/or medial torsion of the proximal tibia and medial displacement of the tibial tuberosity are some deformities which can cause medial displacement of the extensor mechanism and result in the development of PL [2,9,10]. One or more of these abnormalities can cause a QA value modification.

Since the points used to trace the lines of the QA are on the ileum, femur and tibia, any anatomic abnormalities which move one of these three points can change the QA value.

The earliest studies on the QA were carried out on groups of dogs of undefined sizes with no differentiation among the wide variety of canine breeds. Furthermore, the normal hindlimbs of unilateral MPL dogs were assessed collectively with the stifles of healthy dogs without MPL [7,8]. To the authors’ knowledge, only one paper has described the measurement of the QA in healthy German Shepherd Dogs [11].

The purpose of this study was to review the medical records and hindlimb radiographs of healthy dogs in order to determine a normal basic QA value, and to focus on the conformation factor (e.g., QA) which may predispose dogs to MPL by assessing small and medium-large size dogs separately.

The authors hypothesized that the basic QA value was different between small and medium-large breeds, and this difference could have been related to the major biomechanical predisposition small dog to PL. Furthermore, the authors assume that the basic QA value of the entire sample investigated was different from the literature findings.

## Materials and methods

In order to carry out this retrospective study, radiographs of the pelvic limbs of 160 normal dogs were collected from October 2014 through May 2016. The records and radiographs were obtained from the archives of the Department of Veterinary Medical Sciences, University of Bologna, Italy.

### Inclusion criteria

After the assessment of the medical records, only the ones related to dogs considered healthy on the basis of clinical evaluation and previous radiological evaluation, without any orthopaedic diseases affecting the hindlimb, especially PL, hip dysplasia, cranial cruciate ligament rupture, or other causes of lameness, were included in the study. No breed, body-weight, age or sex restrictions were introduced into the inclusion criteria. In terms of quality and positioning, the radiographs of the pelvic limbs of these dogs were coherent with the criteria of the radiographic study described below. The medical records included the informed consent form signed by every dog owner as required for procedures involving anaesthesia.

### Radiographic study

The radiographs of the pelvic limbs were obtained from dogs under deep sedation using dexmedetomidine (Dexdomitor, Orion Corporation, Finland) 3–5 μg/kg, buthorphanol (Alvegesic, Dechra, Italy) administered intramuscularly, and propofol (Proposure 1%, Merial Italy) 1 mg/kg, administered intravenously. The pelvic limbs were extended caudally and rotated internally so that the femurs were parallel, and each patella was centered in the trochlear groove of its respective femur with a symmetrical pelvis, including the proximal tibia [12].

The radiographic images were taken using an FCR Capsula (Fujifilm, Italia). The radiographic measurements of the QA were carried out using a computer program for image analysis (DIGIMIZER®, MedCalcSoftware Ltd, Mariakerke, Belgium).

**Fig 1:**
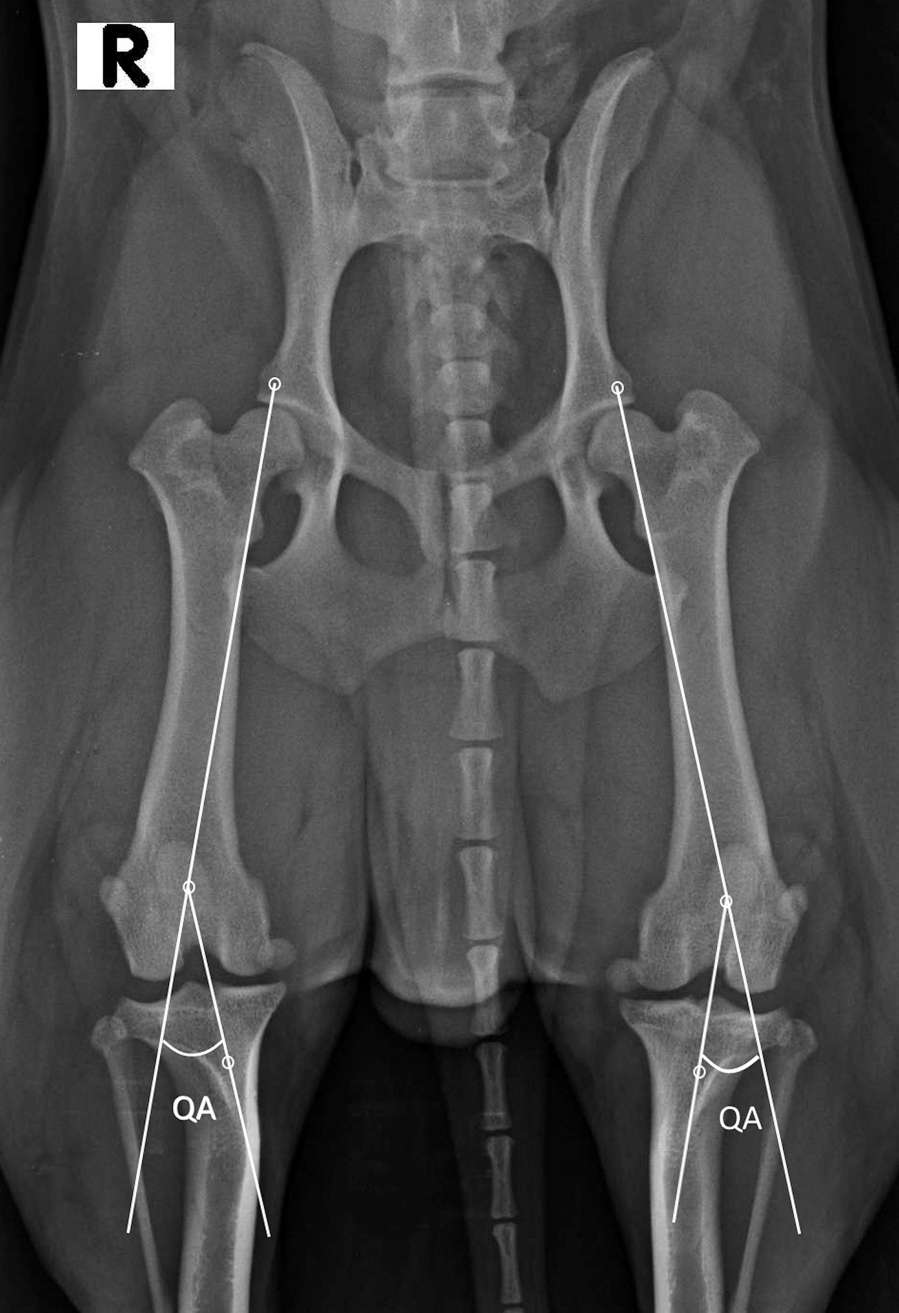
Reference lines for measurement of the quadriceps angle. The QA was measured between the line drawn from the origin of the rectus femoris on the ventral aspect of the ilium, corresponding to the cranial margin of the acetabulum, passing through the middle of the femoral trochlea, and a second line drawn from the middle of the trochlea to the tibial tuberosity. The cranio-caudal radiograph of a 13-month-old male West Highland White Terrier, 9.4 kg body weight; the medical report did not reveal lameness, patella luxation and/or cruciate ligament rupture. The QA values were 22.61° on the right and 21.84° on the left.

The QA was measured as described by Kaiser [8]. The first line was drawn from the origin of the rectus femoris in the area musculi recti femoris of the ileum on the ventral aspect of the ilium, corresponding to the cranial margin of the acetabulum, and passing through the middle of the femoral trochlea, and a second line was drawn from the middle of the femoral trochlea to the tibial tuberosity (Fig 1) [8,13–16]. The middle of the trochlea corresponded to the centre of the patella when it was in its anatomic position, such as in a normal limb and when the radiograph was performed correctly [17]. All measurements were performed by SP, Diagnostic Imaging PhD, who was the same operator who evaluated each radiograph to avoid inter-observer variation errors [18].

### Statistical analysis

Breed, body weight (BW) and gender data were collected. The medians and ranges of the QA were calculated for each right and left pelvic limb. The QA values were calculated for the entire sample: the animals were then divided into two groups: dogs with a weight inferior to 15 kg (Group A) and dogs with a weight superior to 15 kg (Group B). Moreover, the interdecile range (IDR) and the intequartile range (IQR) were calculated in order to report the distribution of the QA value around the median. All the QA values obtained were analysed and the distribution of the data appeared not to be normal in both Group A and Group B, based on the result of Levene’s Test; the non-parametric Mann-Whitney Test was then used. The data in each group were compared among the different breeds, weight and gender of the animals.

All the statistical analyses were a carried out using commercial software (MedCalc® Software 16.8.4, Ostend, Belgium) and significance was set at p<0.05.

## Results

The hindlimbs of each dog were considered separately for a total of 320 normal stifles, and the median QA value in the sample was measured to be 12.4° (range 2.7°-29.7°). Significant differences were not found between the right (n.160) and left (n.160) hindlimbs (p = 0.2649). The medical records of 160 healthy dogs were assessed and divided into two groups.

The dogs were divided into groups based on BW: <15 kg and >15 kg (Table 1). In order to have more details in relation to the influence of the weight, the group >15 kg was divided into an additional two groups with a cut-off value of 30 kg BW, but the difference was not significant (p = 0.7594).

**Table 1:** Distribution of the breeds in Group A (< 15 kg) and Group B (> 15 kg).

Group A		Group B	
Breeds	n. dogs	Breeds	n. dogs
American cocker spaniel	2	Amstaff	2
Beagle	9	Bernese Mountain Dog	6
Chihuahua	6	Border Collies	6
French Bulldog	2	Cane Corso Dog	3
Jack Russell Terrier	14	Czechoslovakian Wolf dog	5
Lagotto Romagnolo dog	1	Doberman	2
Maltese	16	English Setter	5
Miniature American Shepherd	1	German Shepherd	9
Podengo Portugue	7	Golden retriever	4
Poodle	7	Greater Swiss Mountain Dogs	4
Pumi	2	Labrador Retriever	22
Pug	2	Newfoundland Dog	2
Spitz	2	Rottweiler	4
West Highland White Terrier	4	Weimaraner	6
Zwergpinscher	5		

Group A (BW <15 kg): 80 dogs with a median weight of 8 kg (range, 1.8–15), 48 males and 32 females, with a median age of 23 months (range, 12–101). The breeds included are listed in Table 1. The Maltese was the most numerous breed in Group A (16 of 80 dogs: 20%).

Group B (BW >15 kg): 80 dogs with a median weight of 32 kg (range, 18–68), 39 males and 41 females, with a median age of 19 months (range, 13–50). The breeds included are listed in Table 1. The Labrador Retriever was the most common breed in Group B (22 of 80 dogs: 27.5%).

The radiographs of 160 hindlimbs/group were evaluated and their QAs measured; the data are summarised in Table 2.

**Table 2:** Statistical details of the QA influenced by body weight, breed, sex and right/left hindlimbs. Median and range, P-value, Mean, Interdecile range and Interquartile range are listed.

Sample	Number of hind limbs	QA-value median and range	Mann-Whitney Test	Mean	Interdecile range	Interquartile range
Right hind limbs	160	11.5° (2.7°-29.7°)	p = 0.2649	13.4°	6.2°-22.2°	8.2°-18.4°
Left hind limbs	160	12.9° (4.8–29.3)		14.0°	6.9°-23.8°	9.0°-18.1°
Group A	160	18.3° (6.1°–29.7°)	p< 0.0001	18.6°	12.8°-25.6°	14.6°-22.2°
Group B	160	8.7° (2.7°-14.8°)		8.8°	5.8°-11.9°	7.2°-10.6°
Maltese	32	16.6° (11.9°-25.6°)	p = 0.1542	17.6°	12.9°-25.3°	13.9°-19.5°
Group A (all the other breeds)	128	18.6° (6.1°-29.7°)		18.9°	12.8°-25.5°	14.7°-22.2°
Labrador R.	44	9.3° (6.4°-14.8°)	p = 0.0005	9.9°	7.5°-13.3°	8.3°-11.3°
Group B (all the other breeds)	116	8.2° (2.7°-14.6°)		8.4°	5.4°-11.3°	6.5°-10.3°
Male in Group A	96	19.3° (10.1°-29.7°)	p = 0.7979	18.8°	12.7°-26.1°	14.0°-22.3°
Female in Group A	64	17.0° (6.1°-29.6°)		18.4°	12.8°-25.6°	14.7°-22.6°
Male in Group B	78	8.8° (4.7° -14.4°)	p = 0.3822	8.6°	5.9°-11.5°	6.8°-10.6°
Female in Group B	82	9.0° (2.7°-14.8°)		9.0°	5.8°-12.2°	7.3°-10.5

Group A: QA = median 18.3° (range 6.1°–29.7°). The QA of the Maltese was 16.6° (range 12.9°–25.6°).

Group B: QA = median 8.7° (range 2.7°–14.8°). The QA of the Labrador Retriever was 9.3° (range 6.4°-14.8°).

**Fig 2:**
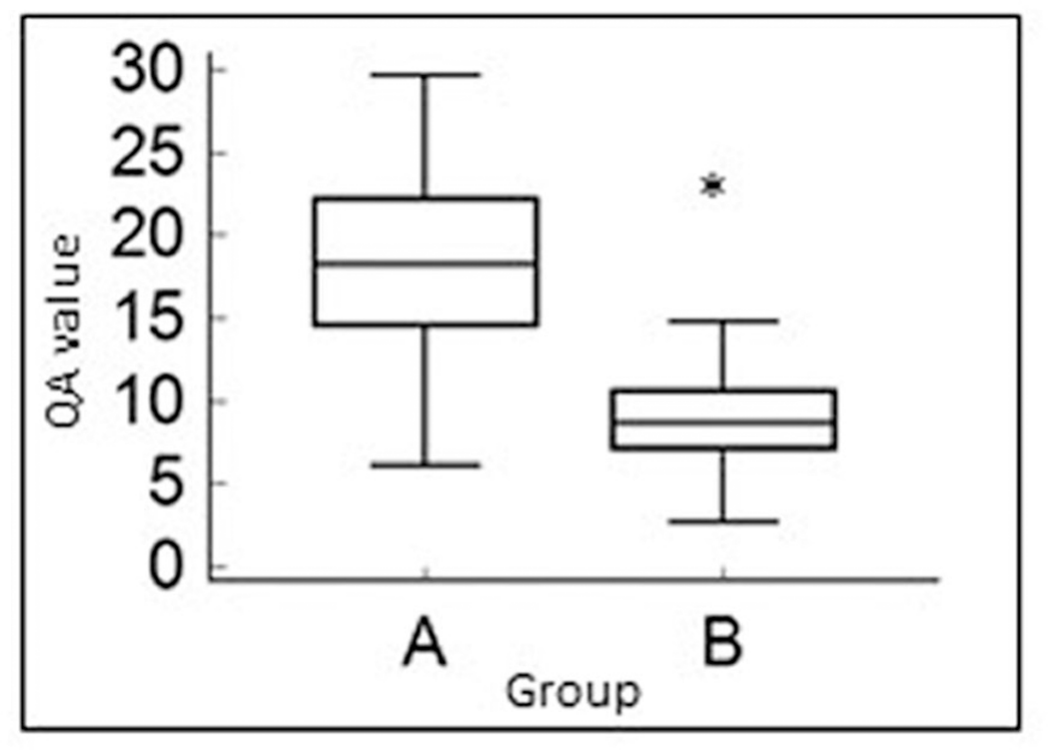
Graphical representations of the median and range of the QA in Groups A and B. *Significant difference of QA values at P < 0.05.

The QA value was significantly higher in Group A than that in Group B (p<0.0001) (Fig 2).

The QA value of the Maltese was not significant in Group A (p = 0.1542); instead, the QA value of the Labrador Retriever was significant in Group B (p = 0.0005). Significant differences between males and females were not found (Table 2).

## Discussion

The results of this study confirmed the authors’ hypothesis: the QA in small animals was statistically different from the QA in medium-large animals. Moreover, the findings obtained from the entire sample, from the small and medium-large dogs (QA = 12.4°, 18.3° and 8.7°, respectively) were in contrast with the QA values found in the literature, referred to as a physiological angle of 10°.

Kaiser et al. in 1997 (42 dogs) and then in 2001 (38 dogs) assessed the QA 10.7°± 4.9° (range 0–26°) of 37 normal pelvic limbs of dogs of different breeds, of different ages and without reporting their BW [7,8]. The QA measurement of those reports were assessed from the limbs of 13 dogs with no orthopaedic problems and from the normal hindlimbs of dogs affected by unilateral MPL. Therefore, the authors concluded that, in the normal group, the QA value of Kaiser’s study may have been influenced by the inconsistency of the sample and by the presence of contralateral stifle pathologies. The QA = 10.7° referred by Kaiser et al. [8] is less than the QA = 18.3° of Group A (<15 kg BW) and greater than the one of 8.7° of Group B (>15 kg) of our study in which the normal QA value was surveyed in only healthy animals.

The normal QA value of Group A (18.3°) in the present study was slightly greater than the QA value measured on 6 small-medium dogs (BW = 3.4–20 kg) with grade II and III MPL (QA = 16.4°±4.9°) described by Towle et al. [16].

The same normal QA value of Group A was intermediate between Mortari’s QA values of 8 dogs with grade I MPL (14.9°±7°) and 8 dogs with grade II MPL (22.1°±6.4°), of all small breeds (3–8 kg) [19]. The measurements were taken manually with a transparent plastic goniometer placed directly on the radiographs in contrast with our digital method.

In light of these comparisons with some results present in the literature, the normal QA value of the present survey is similar to the QA value of dogs with MPL. Therefore, the authors hypothesised that the higher normal QA values of the healthy dogs in Group A could have been due to the bone structure conformation in small dogs, known to be a predisposing factor for the development of PL [1–3].

In Group A, the most common breed was the Maltese (20%). Its QA value (16.6°, range 11.9°-25.6°; IDR 12.9°-25.3°; IQR 13.9°-19.5°) was consistent with the QA of the others in Group A and, therefore, it did not influence the value of the group (p = 0.1542). According to the literature, this breed is one of the miniature breeds which has a high predisposition to MPL [1,2].

The Labrador Retriever was the most common breed in Group B (27.5%). Its QA value (9.3°, range 6.4°-14.8°; IDR 7.5°-13.3°; IQR 8.3°-11.3°) was only one degree higher than the QA of the others in the group, despite the fact that it was statistically significant (p = 0.0005). The highest value of the range of Group B (14.8°) was reached by this breed. This finding is in accordance with Gibson et al. who investigated 70 large dogs with PL, 25 dogs of which were Labrador Retrievers, leading them to evaluate conclude that this breed was at high risk of MPL [20]. Instead, the other breeds of this group did not report significant predispositions to PL [1].

Although the QA value of the Labrador Retriever was considered to be statistically significant as compared to the QA value of Group B, the measurement ranges widely overlapped and the evaluation of the IDR and the IQR still revealed overlapping, reducing the power of the statistical Mann-Whitney test.

Instead, even though the QA ranges obtained from the investigation of both Groups A and B overlapped, the calculation of the IDR and the IQR eliminated the overlapping data in the QA range (Table 2).

The present study was conducted on healthy dogs in order to avoid the presence of QA alterations; animals with skeletal hindlimb abnormalities could have developed PL, a condition which could induce an increase in the QA value if compared to the physiological angle [2,7–10,16,19]. All the dogs included had to have had a hindlimb radiographic image previously analysed in order to have been included this study. The medical records reported the lack of any orthopaedic diseases, such as PL, hip dysplasia, cranial cruciate ligament rupture, or any other causes of hindlimb lameness on the basis of clinical evaluation.

The radiographs showed normal hips and most of them were also certified as “no dysplastic” by Italian board-certified veterinary radiologists. All the radiographs were taken in the ventrodorsal projection, which did not suitable to evaluate the anteversion of the femoral neck that requires the axial view of the femur [12,21]. Therefore the present study is lacking of the data on the anteversion angle.

The literature has produced discordant studies regarding the changes of the anteversion angle of the femoral neck which can influence patellar instability; therefore, additional studies should be carried out to determinate the effect of femoral neck anteversion on the QA [22–24].

The results of Lojszczyk-Szczepaniak’s study, obtained from healthy dogs, were higher than those in Group B, being 17° vs. 8.7° respectively, but the landmark of the apex QA used to calculate the angle was different from Kaiser’s report [7,8,11]. The landmark of the apex was in the centre of the intercondylar notch rather than at the middle of the femoral trochlea as Kaiser described; that landmark is located closer to the tibial tuberosity resulting in higher values of the QA [11,17].

The QA may be calculated in clinical practice using radiological tools, as the accuracy of x-ray examinations was compared to magnetic resonance measurements yielding similar values: QA 10.7° vs. 10.5° respectively [8].

Moreover, the difficulties in identifying the landmark of the tibial tuberosity could have been the reason for the discrepancy of values when compared with those reported in the literature; additional surveys should be carried out to eliminate measurement errors.

The proximal marker identification of the origin of the rectus femoris is not radiologically visible; consequently, it could be subjected to bias. On the radiographic image, the origin of the rectus femoris likely corresponds at the ventral aspect of the ilium on the cranial margin of the acetabulum. A thorough knowledge of the radiographic anatomy of the muscle attachment is imperative. Anatomical and radiological studies associated with an accurate experience are required to calculate the QA [13–15].

The measurement of the QA is not an objective parameter to diagnose or grade PL since this may be assessed solely by orthopaedic examination, but it could reveal the amount of deviation of the quadriceps force when compared to the normal basic QA value.

In the present study, the lack of an MPL group is due to the authors’ aim of identifying the QA range in healthy small and medium-large breeds of dogs. An additional investigation should be carried out to compare these data with those obtained in groups of animals with orthopaedic diseases and to calculate the predictive value to use in clinical practice.

Additional epidemiological studies should be undertaken to compare the literature findings with those based upon the current development and geographical distribution of various breeds, and to find a relationship between the QA value and the deformities which can cause medial displacement of the extensor mechanism in dogs with PL.

## Conclusions

In conclusion, in the present study a significant QA difference was found among the two groups (Group A = 18.3° vs Group B = 8.7°) of normal dogs.

The normal QA value obtained from the small dogs, compared to the literature, is greater than the QA value of normal dogs and is similar to that of dogs with MPL. The QA represents the force generated by the quadriceps muscle which increases in dogs with MPL [8]. Therefore, the authors hypothesised that the QA = 18.3° of normal animals may be an objective parameter to explain the major biomechanical predisposition of small dogs to PL, and a likely predictor of PL appearance in the course of their growth.
